# Targeting Aging: Lessons Learned From Immunometabolism and Cellular Senescence

**DOI:** 10.3389/fimmu.2021.714742

**Published:** 2021-07-21

**Authors:** Dominique E. Martin, Blake L. Torrance, Laura Haynes, Jenna M. Bartley

**Affiliations:** Center on Aging and Department of Immunology, University of Connecticut School of Medicine, Farmington, CT, United States

**Keywords:** immunometabolism, geroscience, aging, T cell, senescence

## Abstract

It is well known that aging is associated with dysregulated metabolism. This is seen both in terms of systemic metabolism, as well as at the cellular level with clear mitochondrial dysfunction. More recently, the importance of cellular metabolism in immune cells, or immunometabolism, has been highlighted as a major modifier of immune cell function. Indeed, T cell activation, differentiation, and effector function partly depend on alterations in metabolic pathways with different cell types and functionality favoring either glycolysis or oxidative phosphorylation. While immune system dysfunction with aging is well described, what remains less elucidated is how the integral networks that control immune cell metabolism are specifically affected by age. In recent years, this significant gap has been identified and work has begun to investigate the various ways immunometabolism could be impacted by both chronological age and age-associated symptoms, such as the systemic accumulation of senescent cells. Here, in this mini-review, we will examine immunometabolism with a focus on T cells, aging, and interventions, such as mTOR modulators and senolytics. This review also covers a timely perspective on how immunometabolism may be an ideal target for immunomodulation with aging.

## Introduction

As humans age, progressive deterioration leads to impaired function and continual decline that results in increased risk of disease and death (1). Aging is characterized by six hallmarks that contribute to this process and determine the aging phenotype. These include genomic instability, telomere attrition, epigenetic alterations, loss of proteostasis, altered intracellular communication, stem cell exhaustion, cellular senescence, mitochondrial dysfunction, and dysregulated nutrient sensing (1). Two hallmarks, mitochondrial dysfunction and dysregulated nutrient sensing, are tightly associated with metabolic alterations. Increasing research is investigating the role of metabolism in controlling longevity (2). In line with the hallmarks of aging, several metabolic changes accumulate with age, indicating the presence of a “metabolic clock” (2).

Three metabolic and nutrient sensing pathways, mechanistic target of rapamycin (mTOR), AMP-activated protein kinase (AMPK), and sirtuins, are under investigation as potential targets for aging (1, 3). mTOR is involved in key cellular processes such as sensing amino acid concentrations, protein synthesis, and autophagy. mTOR is essential for maintaining physiological and cellular homeostasis, and its dysregulation is associated with various metabolic disorders, cancer, and aging (4). mTOR kinase is involved with two complexes, mTORC1 and mTORC2, that modulate most aspects of anabolic metabolism (5). mTOR activity is associated with aging and longevity and is an attractive investigational target in the aging field (6, 7). AMPK detects low energy states by sensing high AMP levels, and sirtuins detect low energy states by sensing high NAD^+^ levels (3). These pathways are involved in detecting nutrient scarcity and catabolism, and have age-associated dysregulation (3). Upregulation of AMPK and sirtuins has been shown to favor healthy aging (3). Additionally, AMPK activation has a multitude of effects on metabolism and healthspan and downregulates mTORC1 (8). Various pathways such as the ones above have all been implicated in dysregulated metabolism with aging and may be useful therapeutic targets for age-related deficits.

Although all systems are affected, the hallmarks of aging substantially impact the immune system. It is well known that aging leads to progressive declines in innate and adaptive immunity (9–12). This immunosenescence is accompanied by chronic low-grade inflammation, or inflammaging (13, 14). This results in increased susceptibility to infections, reduced response to vaccination, and increased prevalence of cancers, autoimmune and chronic diseases. These deficits are reviewed in detail elsewhere (9–12). Not surprisingly, given that deregulated nutrient sensing and mitochondrial dysfunction are hallmarks of aging (1), markers of inflammaging also coincide with markers of metabolic dysfunction (13). Recent research has highlighted the importance of mitochondrial function and cellular metabolism in controlling immune cell function. Indeed, immunometabolism is critical for proper immune function (15–17). What remains to be fully explored is how age and age-associated factors, such as senescent cell accumulation, impact immunometabolism and therefore immune function. This research gap represents a potentially fruitful target for immune modulation in older adults. Here in this mini-review, we will provide an overview of aging, senescence, and immunometabolism with a focus on T cells and interventions, such as mTOR modulators and senolytics.

## Immunometabolism

Metabolic control of the immune system provides insight into cellular mechanisms influencing immune cell fate and function. Six major metabolic pathways including glycolysis, fatty acid oxidation (FAO), fatty acid synthesis, tricarboxylic acid (TCA) cycle, amino acid metabolism, and the pentose phosphate pathway are involved in controlling immune cell metabolism (15–17). Cell intrinsic and extrinsic signals modulate the activity of immune cells by altering metabolism. Metabolic signaling helps regulate growth, proliferation, and survival needs of immune cells while also influencing effector function. Immune cell subtypes adopt distinct metabolic phenotypes to balance energy requirements, biosynthesis demands, and longevity. During an immune response, immune cells must become activated and change their functional activities in order to respond appropriately (16). Additionally, immune cells must traverse multiple tissues and microenvironments with varying oxygen levels and nutrient availability (18). Thus, immune cells face different metabolic requirements based on their state and tissue environment.

Generally, resting immune cells are relatively inert, but undergo distinct metabolic reprogramming in response to pathogens. Pro-inflammatory immune cells are engaged in aerobic glycolysis where glucose is metabolized into lactate in the presence of oxygen. This glycolytic phenotype is necessary for actively proliferating immune cells to provide biosynthetic precursors essential for synthesis of amino acids, nucleotides, and lipids (19). Additionally, aerobic glycolysis provides carbon intermediates that feed into other biosynthetic pathways and supplies components necessary to sustain proliferation and production of inflammatory molecules (20). Cells with a glycolytic signature can also adapt to restrictive conditions like hypoxia (20). Conversely, immune cells can also utilize the TCA cycle and oxidative phosphorylation (OXPHOS). This pathway is generally employed by immune cells that are quiescent or non-proliferative whose main requirements are energy and longevity (18). In actuality, immune cell subsets use a combination of OXPHOS, glycolysis, and aerobic glycolysis to regulate immune function. The metabolism of many specific immune cell subsets has been previously reviewed (16, 18) and is beyond the scope of this mini-review. In short, metabolism plays multiple roles in various immune cells to regulate immune cell function.

## T Cell Immunometabolism

T cells are particularly influenced by metabolic signaling. T cells develop and mature in the thymus and then enter circulation as naïve T cells (T_naive_). Once stimulated with antigen, T_naive_ differentiate into effector T (T_eff_) cells and memory T (T_m_) cells. The transition from T_naive_ to T_eff_ to T_mem_ cells requires distinct metabolic coordination. The metabolic events surrounding the coordination of T cell quiescence and activation have been reviewed previously (21, 22). Initially, T_naive_ are mostly metabolically inert and generate energy through OXPHOS which is suited for their resting lifestyle. Specifically, T_naive_ primarily rely on glucose-derived pyruvate from OXPHOS, or utilize FAO (23–26). During T cell activation, there is metabolic reprogramming of antigen specific T cells which have been stimulated *via* T cell receptor (TCR) ligation and binding of costimulatory molecules. The switch from T_naive_ to an activated T_eff_ is characterized by an overall shift in metabolic programing favoring glycolysis over OXPHOS (20). Despite being less efficient than OXPHOS at generating ATP, aerobic glycolysis allows for generation of metabolic intermediates necessary for proliferation and assists in maintaining redox balance (20).

Additionally, CD4 T helper (Th) cell subset differentiation and function is highly dependent on metabolism. Alterations in glycolytic metabolism and mTOR signaling promote differentiation of Th cells into Th1, Th2, Th17, or T regulatory (Treg) cells (27–29). Tregs depend more FAO, whereas Th1, Th2, and Th17 cells strongly activate glycolysis through mTOR signaling (29). Changes to T_eff_ cell metabolism, in both the CD4 and CD8 compartments, are critical for the transition of T_eff_ to T_mem_, which are generally quiescent and rely on OXPHOS. Subsequently, following antigen challenge, T_mem_ increase both OXPHOS and glycolysis to facilitate recall responses (30–32).

## Aging and T Cell Immunometabolism

Metabolic alterations are closely linked to the changes seen in various immune cell populations, including T cells, with aging. This can result in impaired function as well as compromised effectiveness when facing infections (33). The effects of aging on T cell immunity are broad and include both T cell intrinsic and extrinsic effects. With aging, T cell populations shift from mainly naïve to memory subsets (34). Additionally, aging is associated with reduced TCR diversity (35). These phenotypic changes result in overall compromised response to new antigen.

It’s likely that changes to T cell metabolism partially drive these phenotypic differences seen with aging since metabolism is a critical component of effective immune responses. Current research supports metabolic differences between young and aged T cells that can alter cellular function. Specifically, CD4 T cells from older adults had higher baseline and maximal oxygen consumption rate, extracellular acidification rate ratio, and proton leak compared with young CD4 T cells (36). Additionally, upon activation, CD4 T cells from aged mice had altered metabolic reprogramming resulting in compromised metabolic pathways such as OXPHOS and glycolysis (37). Other studies demonstrated metabolic differences such as altered spare respiratory capacity between young and aged CD8 T cells (38). The mechanisms driving metabolic differences in aging T cells are not fully understood, but current research is investigating metabolic profiles in aged T cell subsets and how this impacts function (39). While the research is limited on direct alterations in immunometabolism in aged T cells, many lines of thought support the idea that metabolic changes with aging affect immunometabolism. Future research will better define these changes and how they can be targeted for immune modulation.

## Cellular Senescence and Immunometabolism

While work focused on immunometabolism has revealed potentially intervenable deficits associated with age, indirect mechanisms that can also contribute to immune dysfunction. Namely, senescent cells have shown to be key players in the exacerbation of various symptoms of aging and likely contribute to declines in immune cell function and optimal immunometabolism. In the past decade, many studies have linked cellular senescence and senescent cell accumulation with age-related phenotypes and diseases. Cellular senescence is a mostly irreversible state of cell cycle arrest that was initially understood as terminal replicative exhaustion (40). Years later, it became apparent that senescent cells contribute to the overall progression of age-related dysfunction at the organismal level. It was also found that various stimuli induce senescence other than replicative exhaustion. Oncogene expression, genotoxic stress, growth factor signaling, and loss of proteostasis can also induce senescence (41). With age, senescent cells accumulate in various tissues and contribute to tissue dysfunction. Accumulation of these cells also causes a systemic switch in metabolism (42). Although proliferation is arrested, senescent cells are very metabolically active. In general, senescent cells are more glycolytic than their non-senescent counterparts (43). Senescent cells require synthesis of a myriad of signaling molecules related to their secretory program, the senescence associate secretory phenotype (SASP). By increasing reliance on glycolysis, senescent cells are able to support production of this complex and destructive SASP.

The SASP is a heterogeneous, generally pro-inflammatory mix of cytokines, chemokines, and other molecules that contribute to the dysfunction caused by senescent cells (44). These factors alter the tissue microenvironments and induce the chronic, basal, and sterile inflammation, i.e., inflammaging, that is seen with age. Senescent cells, through the SASP, can signal in a paracrine matter to induce senescence in adjacent cells (45). This accumulation of senescent cells will then not only increase the deleterious effects of the SASP but also cause a significant shift in metabolic programming within the tissues (46). Because senescent cells rely heavily on glycolytic pathways, this results in dramatic changes to metabolite utilization and can cause redox stress, further inducing senescence within local environments (47). Although little has been done to assess how this affects the function of immune cells, it is likely that the accumulation of senescent cells and this shift in metabolism negatively impacts immune responses. Indeed, the effect of many different SASP components have been shown to have individual effects on immune cell fate and function (48).

As noted, proper T cell function requires complex orchestration of metabolic switching. While it is controversial if T cells themselves become truly senescent since they tend to lose the ability to secrete cytokines (48–52), it is clear that with age, T cells have lower proliferative capacity and altered functionality (48–50, 53, 54). Many of the mechanisms that are involved with coordinating their functionality are altered with exposure to SASP factors (46). mTOR is highly active in senescent cells for SASP production (55, 56). Aberrant mTOR activation therefore can be described as an underlying cause and symptom of systemic dysfunction wrought by the accumulation of senescent cells and SASP.

Nicotinamide adenine dinucleotide (NAD^+^) is another key metabolite that has dramatic changes with age. NAD^+^ participates in many processes of metabolic homeostasis, including redox reaction steps in nearly every major metabolic pathway. Glycolysis, TCA cycle, OXPHOS, and FAO all include NAD^+^. With age, however, systemic levels of NAD^+^ decline and have been linked to many of the hallmarks of aging. Aging causes increased activity of NAD^+^ consuming pathways including poly (ADP-ribose) polymerases (PARP) mediated DNA-damage repair (57) and sirtuin activity (58), which drive this systemic decrease. CD38, a cell-surface glycoprotein that consumes NAD^+^ to generate ADP ribose, has emerged as the key regulator of NAD^+^ decline *via* a sirtuin-dependent pathway (59). CD38 is highly expressed in a variety of immune cells and participation of immune cells in this paradigm is emerging. Recently, it was shown that culturing macrophages with SASP factors upregulated CD38 expression, which caused a marked decline in systemic NAD^+^ levels (60). Complicating this paradigm, NAD^+^ has also been shown to reinforce the secretome of senescent cells *via* AMPK signaling (61). Aside from the direct role played by macrophages in mediating the decline of NAD^+^, the effects of this paucity on adaptive immune function have yet to be fully explored. T_eff_ rely heavily on NAD^+^ to support glutaminolysis and glycolysis, thus NAD^+^ scarcity could play a large role in T cell functional deficits with age.

## Discussion

Interventions that target dysregulated metabolism or senescence may prove fruitful for improving aged immune responses, leading to additional protection when dealing with infection. In fact, Mannick et al. (62) demonstrated that low dose TORC1 inhibition in older adults decreased risk of all infections, upregulated antiviral immunity, and improved influenza vaccination responses. This work confirmed that metabolism is a valuable target to improve immune responses in older adults. Current research is investigating how metabolic regulators affect T cell function and metabolism with aging. Indeed, metformin, an FDA approved diabetes drug that modulates mTOR/AMPK has been investigated as a potential therapeutic to target deficits with T cell aging. Metformin enhances T cell autophagy, normalizes mitochondrial function, and alleviates aging-associated Th17 inflammation (36). Similar to rapamycin, metformin extends healthspan and lifespan in multiple animal models (63–66). Additionally, in young mice, metformin can increase CD8 T cell memory formation through AMPK activation and FAO enhancement (31). Although further research is necessary to fully elucidate the mechanisms by which metabolic changes with aging contribute to T cell dysfunction, current metabolic therapeutics may prove beneficial for targeting the aging immune system.

Furthermore, targeting senescent cells may also improve immunometabolism with aging. Senolytics, drugs that target senescent cells, have shown great promise with treating age-related diseases and phenotypes. Treatment with the combination of dasatinib and quercetin (D+Q) is the first described and most well characterized senolytic (67) and improves both life- and healthspan in mice (68). Also in mice, D+Q has treated idiopathic pulmonary fibrosis (69), non-alcoholic fatty liver disease (70), and type 2 diabetes (T2D) (42). In humans, clinical trials have shown efficacy in clearing senescent cells while alleviating symptoms of diabetic kidney disease (71) and idiopathic pulmonary fibrosis (72). Trials are underway utilizing D+Q in other contexts as well as investigating the use of other senolytics, including fisetin. Studies directly linking senolytics and metabolism have been mostly limited to alleviation of T2D. With age, there is increased insulin resistance driven by dysfunction in adipose tissue (73). Senescent adipocyte progenitor cells were found to be the root cause of dysfunction and when cleared with D+Q insulin resistance is reversed (42). As with most senescence associated phenotypes, SASP is the means by which insulin resistance is likely conferred (74). Interestingly, many immune cells (including T cells, B cells, and NKT cells), are thought to exacerbate insulin resistance (75). Taken together, the ablation of SASP using senolytics could possibly reverse the deleterious activity of these immune cells which could be beneficial beyond insulin resistance. Furthermore, senolytics may reduce CD38 expressing macrophages and preserve NAD^+^ levels with aging, offering a promising strategy to enhance metabolic fitness with age.

Similarly, targeting the SASP directly or indirectly *via* mitigation of SASP effects may be a valuable way to improve immunometabolism and immune function. Augmentation of NAD^+^ *via* supplementation or other indirect methods may improve aging metabolism. Indeed, supplementation studies to increase NAD^+^ to improve different aspects of metabolism and healthspan show tremendous promise (76–78), however the effect on immunometabolism and immune function requires further study. Similarly, CD38 inhibitors *via* anti-CD38 antibodies, NAD analogs, flavonoids, or others, have shown promise in tumor immunity, aging, and metabolic diseases (79–81). More specifically, thiazoloquin(az)olin(on)e CD38 inhibitor can reverse age-related NAD+ decline and improve both functional and metabolic aspects of aging (79). However, its effect on immune function requires further investigation.

Certainly, given the impact of senescent cells on metabolic function and the vast amount of metabolic dysfunction with aging, it is possible that senolytics can modulate immunometabolism and ameliorate age-related immune decline in the context of infection or even vaccination. This strategy is being currently investigated to improve COVID-19 outcomes in an ongoing clinical trial investigating the use of fisetin to mitigate COVID-19 induced cytokine storm in hospitalized patients (NCT04476953). Another group is targeting metabolism *via* metformin to improve illness in non-hospitalized COVID-19 patients (NCT04510194). Thus, ongoing research will better define the utility of senolytics and metabolic modulators to improve immunometabolism and immune function in the face of infection.

Non-pharmaceutical alternatives, mainly physical exercise and caloric restriction, have been shown to target many of the hallmarks of aging (82–84). Exercise prevents diet-induced cellular senescence (85, 86) and many facets of immunosenescence (87). Similarly, caloric restriction extends lifespan and healthspan in multiple species (83, 84, 88), improves metabolism (89), reduces T cell immunosenescence (90), and reduces markers of senescence (91, 92). Thus, while pharmaceuticals are still under development, exercise and caloric restriction are well-supported modulators to improve age-related metabolic and immune dysfunction.

## Conclusions

An immune cell’s ability to modulate its metabolism is intimately linked to optimal function. However, this can become compromised due directly to chronological age or indirectly by consequences of age such as accumulation of senescent cells. While there remains much to be uncovered regarding the mechanisms that connect these multifaceted processes, metabolism and senescence have been shown to be intervenable to ameliorate general age-related diseases. Considering how integral the pathways regulating metabolism and senescence are to immune cell function, they are emerging as attractive and efficacious targets. This creates a new possible point of convergence between the worlds of aging, metabolomics, cellular senescence, and immunology that could be of great importance when pursuing strategies to promote healthy aging and a resilient immune system throughout life.

## Author Contributions

DM and BT wrote the manuscript. LH and JB helped write and critically reviewed the manuscript. JB finalized the manuscript. All authors contributed to the article and approved the submitted version.

## Funding

This work was funded by R21AG060389 (LH) and R21AG060707 (LH).

## Conflict of Interest

The authors declare that the research was conducted in the absence of any commercial or financial relationships that could be construed as a potential conflict of interest.
